# Gastrointestinal Disorders and Food Selectivity: Relationship with Sleep and Challenging Behavior in Children with Autism Spectrum Disorder

**DOI:** 10.3390/children10020253

**Published:** 2023-01-30

**Authors:** Giulia Bresciani, Prisca Da Lozzo, Sara Lega, Matteo Bramuzzo, Grazia Di Leo, Andrea Dissegna, Vissia Colonna, Egidio Barbi, Marco Carrozzi, Raffaella Devescovi

**Affiliations:** 1 Division of Child Neurology and Psychiatry, Institute for Maternal and Child Health—IRCCS “Burlo Garofolo”, 34137 Trieste, Italy; 2 Department of Medical, Surgical and Health Sciences, University of Trieste, 38122 Trieste, Italy; 3 Institute for Maternal and Child Health—IRCCS “Burlo Garofolo”, 34137 Trieste, Italy; 4 Department of Life Sciences, University of Trieste, 38122 Trieste, Italy; 5 CIMeC Centre for Mind/Brain Sciences, University of Trento, 38122 Rovereto, Italy

**Keywords:** autism spectrum disorder, behavioral issues, food selectivity, gastrointestinal symptoms, multidisciplinary approach, parental stress, sleep problems

## Abstract

The aim of this study was to evaluate the interaction between gastrointestinal (GI) disorders, sleep problems, and challenging behaviors in children with a diagnosis of Autism Spectrum Disorder (ASD) and their effect on parental stress. The secondary objective was to assess the frequency and type of GI and feeding disorders in a sample of children with ASD through a multidisciplinary assessment and, finally, to investigate families’ perceptions and satisfaction with the proposed multidisciplinary approach. All children underwent a comprehensive gastroenterological and neuropsychiatric evaluation supported by standardized questionnaires. Pediatric gastroenterologists, specifically trained in Applied Behavior Analysis (ABA), provided advice for parent-delivered behavioral intervention for food selectivity. Thirty-six children with an autism diagnosis (29 males, age 4.5 +/−2.2 years, mean +/− SD) were enrolled. A positive correlation between sleep problems and aggressive behavior was found, and this association was stronger in children experiencing more problematic mealtime behaviors (b = 0.788, *p* = 0.014). Sleep difficulties were associated with stereotyped behaviors and parent-perceived stress. Parents interviewed about the gastroenterology visit perceived this multidisciplinary approach as helpful in addressing food selectivity. This study shows that sleep and mealtime issues can have a synergistic negative impact on ASD symptoms. A multidisciplinary approach and an integrated assessment of GI, feeding problems, and sleep disorders could be helpful in diagnosing comorbidities and to provide targeted advice to parents.

## 1. Introduction

Autism Spectrum Disorder (ASD) is a neurodevelopmental disorder characterized by persistent social communication difficulties, restricted interests, repetitive activities, and sensory abnormalities [1]. These core behavioral features can be associated with many co-occurring medical conditions such as anxiety disorders [2,3], sleep abnormalities [4,5], and gastrointestinal dysfunction, including gastrointestinal (GI) symptoms [6,7] and feeding disorders [8]. Comorbidities can vary in incidence and severity, contributing to the heterogeneity of clinical manifestations in children with ASD [9].

The median prevalence of GI symptoms among children on the autism spectrum is about 46% [10,11], three times higher than their typically developing peers [12,13]. The most reported GI disorders in children with ASD are functional constipation and abdominal pain with or without diarrhea and encopresis [7,14,15]. Multiple GI symptoms are reported by 30.6% of ASD children vs. 5.4% of healthy peers [16]. Food selectivity (FS), including food refusal, disruptive mealtime behaviors, limited food repertoire, and restricted dietary intake, affects as many as 95% of children with ASD [17,18,19]. In these patients, feeding and GI disorders have been associated with multiple challenging behaviors, including anxiety, affective problems, and aggressive behaviors [13,20]. Moreover, a high prevalence of sleep problems in children and GI symptoms has been reported [21,22,23], negatively impacting the quality of life for both the child and the parents [9,24].

However, as reviewed by Mannion and Leader [20], the prevalence of gastrointestinal complaints in children with autism diagnosis may vary greatly, ranging between 9% and 91%, likely due to differences in the populations studied, data collection methods, and definitions of symptoms. Previous studies on the prevalence of gastrointestinal disorders in children with a diagnosis of ASD have some limitations, such as relying solely on parental reports from questionnaires or only including children with recorded symptoms in the previous three months, thus leading to potential underestimation or overestimation of GI symptoms. [25,26,27].

Studies on this topic are important for several reasons, including the high prevalence of symptoms, the discomfort they can cause, and the importance of identifying them in nonverbal or poorly communicative children. Furthermore, it has been recognized that abdominal pain or discomfort may also contribute to behavioral problems, as noted in the work of Buie et al. [10].

A clinical evaluation performed by a pediatric gastroenterologist, integrating data from medical history, parents’ interviews, physical examination, and laboratory tests, should be the gold standard to diagnose both organic and functional GI disorders in children [28]. However, to the best of our knowledge, only one previous study [15] assessed GI disorders in children with an autism diagnosis through a systematic clinical evaluation performed by a pediatric gastroenterologist.

Another poorly explored issue is the effect of the concurrence of GI symptoms and sleep disorders. These two aspects have generally been considered separate, leaving a gap in knowledge about the clinical implications of their potential interaction.

In this study, we sought to analyze how GI and/or feeding disorders and sleep difficulties potentiate each other when concurrently present and how these variables affect challenging behaviors and parental stress. We also systematically investigated the presence of GI disorders and FS through a gastroenterological assessment that we incorporated into our clinical practice. Finally, we assessed parental satisfaction and parent perception of the effectiveness of this multidisciplinary approach.

## 2. Materials and Methods

This was a single-center, observational study carried out according to the standards for the good ethical practice of the university hospital and following the Declaration of Helsinki guidelines. The Study Protocol was approved by the Institutional Review Board (n° 34/18 on 26 August 2019). From May 2020 to May 2021, we consecutively enrolled all children with an autism diagnosis admitted to the Division of Child Neurology and Psychiatry at the University pediatric hospital “IRCCS Burlo Garofolo” for a first clinical evaluation or a follow-up visit.

**Figure 1:**
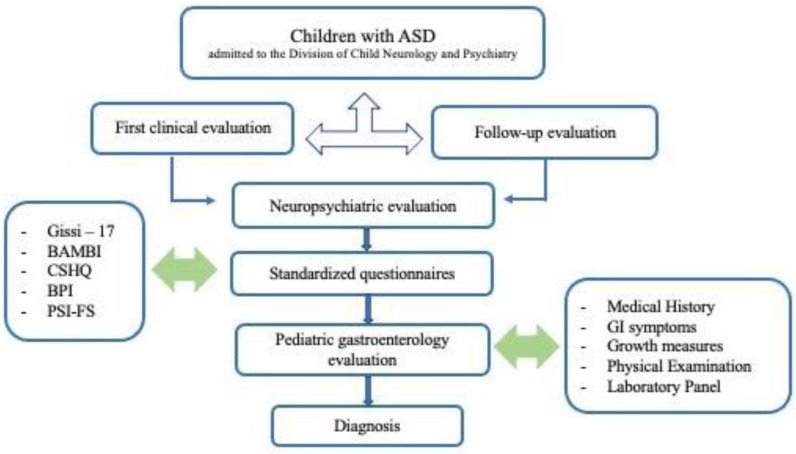
This represents the study’s workflow.

Inclusion criteria were: (1) children who met ASD diagnostic criteria according to the DSM-5 [1] and met a cut-off for ASD on the Autism Diagnostic Observation Schedule—Second Edition (ADOS-2) [29,30] administered by experienced clinicians. Exclusion criteria included psychotropic medication started in the previous six months to avoid possible bias on the behavioral issues. (Figure 1)

All the participants underwent a multidisciplinary assessment, including a neuropsychiatric evaluation performed by a child neuropsychiatrist and a comprehensive clinical evaluation performed by a pediatric gastroenterologist. The latter included medical history, GI symptoms, growth measures, physical examination, a laboratory panel including complete blood count with differentials, a comprehensive metabolic panel with electrolytes and liver function tests, erythrocyte sedimentation rate, ferritin, and celiac screening. Overweight was defined as a BMI percentile at or above the 85th percentile, obesity was defined as a BMI percentile at or above the 95th percentile, and underweight was defined as a BMI percentile below the 5th percentile [31]. As needed, functional constipation was diagnosed and treated according to the current ESPGHAN guidelines [32] with oral polyethylene glycol (PEG).

Specific standardized, validated questionnaires were collected at baseline to support the medical history. In particular, (FS) was specifically investigated and classified as proposed by Mazzone et al. [33] as follows: (a) mild, if less than 15 food items were consumed, at least three food items for each food group (i.e., fruit, vegetable, protein, grain, dairy); (b) moderate, if less than three food items for the food group were consumed; (c) and severe, if less than three food items were consumed weekly. FS was treated with a multi-step approach, including a parent-mediated behavioral intervention for all the patients and a trial with an appetite stimulant (oral cyproheptadine) in moderate to severe cases with poor food intake and nutritional supplements in severe cases [34].

Pediatric gastroenterologists received specific training inspired by the Applied Behavioral Analysis (ABA) principles before the beginning of the study. They became skilled in using principal behavioral techniques indicated for managing FS and mealtime behavior correlated. These techniques include positive reinforcement, escape extinction, differential positive reinforcement, non-contingent positive reinforcement, and stimulus fading. The training was conducted by a certified psychologist and psychotherapist, Board Certified Behavior Analyst (BCBA) and consisted of two days of intensive training followed by four consecutive supervised sessions.

The aim of the training was to empower the pediatric gastroenterologist to recognize the contextual factors, such as the environment, mode of meal presentation, and mode of parental response to challenging behaviors. Furthermore, the training was useful for analyzing mealtime behaviors and providing parents advice on mealtime behavior management in the home setting. This kind of approach has proven successful, mainly in cases of mild selectivity [35].

### 2.1. Measures

To diagnose ASD, we used the Autism Diagnostic Observation Schedule—Second Edition (ADOS-2). The ADOS-2 [29,30] is a standardized diagnostic observational instrument that assesses restrictive behavior, play, social reciprocity, and communication as ASD signs. We used the ADOS calibrated severity score [36], which enables comparison across several ADOS modules, to compare the severity of ASD among participants. Using the methods outlined by Hus et al. [37] and Esler et al. [38], we estimated the Calibrated Severity Scores Overall (CSS Overall), CSS for the Social Affect algorithm (CSS SA), and CSS for the Restricted and Repetitive Behavior algorithm (CSS RRB).

To assess the frequency and type of gastrointestinal disorders, we used:

₋ GISSI-17 (AS-ATN GI Signs and Symptoms Inventory-17): a 17-items screening questionnaire that targets functional constipation, functional diarrhea, and Gastro-Esophageal Reflux Disease (GERD) [39]
₋ BAMBI (Brief Autism Mealtime Behavior Inventory): an 18-items parent-report questionnaire designed to capture mealtime behaviors specific to children with ASD. Responses score on a 1–5 Likert scale with a score of 1 indicating the behavior “never occurs” and a score of 5 indicating the behavior “always happens” at mealtime. Reversed scoring is used for four of the item’s rating positive mealtime behaviors. A total score of above 34 is the optimal clinical cut-off [40].

To identify sleep and behavior disorders and parental stress, we used the following questionnaires:

₋ CSHQ (Children Sleep Habits Questionnaire): The CSHQ was used to assess sleep disorders in children. It describes eight dimensions: bedtime resistance, sleep onset delay, sleep duration, sleep anxiety, night waking, parasomnias, sleep-disordered breathing, and daytime sleepiness. A total CSHQ score ≥41 was considered a diagnostic of the presence of a sleep disorder. We used a modified CSHQ questionnaire specific for ASD children, as reported by Katz, translated into Italian with a back translation method [41].
₋ BPI (Behavior Problem Inventory): an informant-based behavior rating tool designed to evaluate maladaptive behaviors. The BPI includes three subscales: Self-Injurious Behavior (15 items), Stereotyped Behavior (25 items), and Aggressive/Destructive Behavior (12 items) [42].
₋ PSI-SF (Parenting Stress Index- Short form): 36-item parent self-report designed to measure parental impressions and difficulties about the parenting role. The PSI consists of three 12-item subscales: Parental Distress (PD) (burdens and limitations in the parenting role); Parent-Child Dysfunctional Interaction (P- CDI) (level of dissatisfaction in the parent-child relationship); and Difficult Child (DC) (child behaviors and characteristics that are challenging for the parent). The total PSI score is an overall parenting stress index [43].

Finally, we performed a telephonic interview to investigate the perception of families regarding the multidisciplinary assessment. The questions were: “How important did you consider having a dedicated visit with the pediatric gastroenterologist?” and “How useful, in the household setting, was the counseling provided by the pediatric gastroenterologist?”. Results were expressed on a 5-point Likert scale. For the first question, the score of “1” corresponded to “not at all important” and the score of “5” to “very important”; regarding the second question, the score of “1” corresponded to “not at all useful” while the score of “5” to “very useful.”

### 2.2. Data Analysis

All analyses were performed in R (R Core Team, [44]). After descriptive statistics, a simple correlation analysis was performed to analyze the relationship between our Measures. After that, PSI and BPI scores were analyzed separately using two linear regression models. Both the models included CSHQ, BAMBI, the BAMBI × CSHQ interaction, and age as predictors. Predictors were z standardized. The BMI and CSS ADOS score were not included in the regression models because they were uncorrelated with all the other variables. As an overall test of the goodness of fit for our regression models, we reported R² and the respective F statistics.

We conducted a sensitivity analysis with G-Power 3.1 [45] on our sample size with α err. Prob. = 0.05, Power (1—β err. Prob.) = 0.80 to establish the Minimal Detectable Effects resulting from our experimental design. These resulted in being in the medium-to-large range with a critical F = 2.92 and an R^2^ = 0.26.

## 3. Results

### 3.1. Patient Population

Thirty-six children (twenty-nine males and seven females) were enrolled in the study. The average age for participants was 4.5 years (+/−2.2 SD, ranging from 1 to 10 years). The socio-demographic characteristics of the sample are described in Table 1.

In total, twenty-nine (80%) had a normal weight, two (5%) subjects were overweight (mean BMI Z-score 1.6 +/− 0.3 SD), and three (8%) were obese (mean BMI Z-score 2.8 +/− 0.5 SD). Two (5%) patients were underweight. Laboratory work-up was available for thirty (83%) patients and showed low ferritin levels in nine cases (30%), mild microcytic hypochromic anemia in two cases (5%), and low vitamin B12 levels in one case; the remaining had normal nutritional values. No cases of celiac disease were identified. Two children were reported to follow a special diet (one lactose-free and one gluten-free and lactose-free).

### 3.2. Frequency and Type of GI Disorders and Food Selectivity

At the gastroenterological evaluation, fourteen diagnoses of functional gastrointestinal disorders were made, thirteen (36%) cases of functional constipation, and one case of rumination syndrome. Based on the GISSI-17 questionnaire, 11 (30.5%) children tested positive for diarrhea, 16 (44.4%) for constipation, and 14 (38.8%) for GERD.

Two patients with constipation were already on treatment with an osmotic laxative with clinical improvement; the remaining 11 children with functional constipation started treatment with an osmotic laxative, and caregivers were provided advice for toilet training.

FS was present in twenty (61%) cases and was classified as mild in twelve cases, moderate in seven cases, and severe in three cases. Eight (22%) children had both constipation and FS. The factors that influenced FS were texture (73%), taste (54%), color (18%), temperature (9%), and smell (4%). Problematic mealtime behavior (i.e., BAMBI above the 34-cutoff score) was present in 29 (80%) cases.

### 3.3. Frequency and Type of Sleep Problems, Challenging Behaviors, and Parental Stress

Regarding sleep disorders, the CSHQ screening questionnaire showed that twenty-nine (71%) patients had sleep problems (CSHQ ≥ 41, mean 45); of these, two were already on treatment with melatonin. On average, parents reported children slept 9.8 h per night.

Results from the BPI questionnaire showed that 23 children (63.8%) exhibited self-injurious behaviors, 35 (97.2%) showed stereotypical behaviors, and 26 (72.2%) exhibited aggressive behaviors.

Regarding the parental stress assessment, 13 (36%) parents had a clinical score (>90th) on the PD scale, 15 (41.6%) had a clinical score on the P-CDI scale, 14 (38.8%) parents had a clinical score on the DC scale; overall 14 (3.8%) parents had a clinically significant score on the total scale.

### 3.4. Correlation Analysis

Table 2 reports the intercorrelations between the continuous variables. CSHQ and BAMBI were positively correlated, revealing that the frequency of negative mealtime behaviors increased with the frequency of sleep disorders. Similarly, the CSHQ score positively correlated with the BPI aggressive score and the BPI stereotyped score, indicating a significant relationship between behavioral problems (aggressive and stereotyped) and sleep disorders. Finally, the CSHQ score positively correlated with the total PSI score, revealing greater parent-perceived stress for children with more sleep disorders.

### 3.5. Linear Regression Models

The linear model, including CSHQ, BAMBI, the BAMBI × CSHQ interaction, and age as predictors, accounted for a significant portion of the variance of the PSI total score (R² = 0.432, F (3, 25) = 4.75, *p* = 0.005). The PSI total score was positively associated with both CSHQ and Age. By contrast, neither the association with BAMBI nor the BAMBI × CSHQ interaction effect was significant (see Table 3). The findings indicated that when children have more sleep difficulties, their parents experience higher stress. Furthermore, parents of older children reported more stress than parents of younger children.

Across the PSI subscales, only the PD score was significantly accounted for by our linear model (R² = 0.425, F (3, 26) = 4.82, *p* = 0.004), further corroborating the detrimental effect of sleep disorders and age of the child on parent-perceived stress.

**Figure 2:**
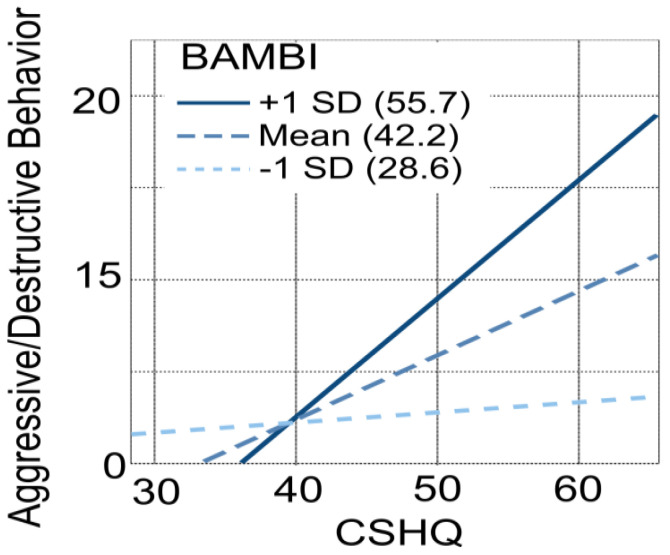
The relationship between sleep disorders (measured through the CSHQ questionnaire) and aggressive behaviors (measured through the BPI questionnaire) is moderated by mealtime behaviors (measured through the BAMBI questionnaire).

The same linear model also accounted for a significant quote of variance of the BPI Aggressive Behavior score (R² = 0.501, F (4, 29) = 7.28, *p* < 0.001, see Table 4). This analysis revealed a significant BAMBI × CSHQ interaction (b = 2.817, s.e. = 1.043, 95% CI [0.681, 4.952]). To obtain a better understanding of this interaction, we estimated the conditional effect of CSHQ on Aggressive Behavior at different levels of BAMBI (1SD below the mean and 1SD above the mean, see Figure 2).

We found that the relationship between Aggressive Behavior score and CSHQ was null at low scores of BAMBI (b = −0.015, s.e. = 0.191, 95% CI [−0.407, 0.376]) but significantly increased at mean (b = 0.387, s.e. = 0.137, 95% CI [0.107, 0.666], *p* = 0.014) and high BAMBI scores (b = 0.788, s.e. = 0.212, 95% CI [0.355, 1.223], *p* = 0.014). This suggests that aggressive behaviors are more common in children with more sleep difficulties, but this association is stronger in children with more negative mealtime experiences. BAMBI, CSHQ, and age did not significantly contribute to determining the frequency of Self-Injurious behaviors (see Table 4). In contrast, there was a significant relationship between Stereotyped behaviors and CSHQ, indicating that children with more sleep difficulties had more stereotypies.

### 3.6. Parental Perception of Integrate Care

The families reported that it was important (Mean = 4.3; SD = 0.84) to have an appointment with the pediatric gastroenterologist and found the counseling useful in the household setting (Mean = 3.3; SD = 1.14).

## 4. Discussion

This study investigates the joint interaction between GI symptoms, feeding disorders, sleep problems, challenging behaviors, and parental stress in a sample of children with ASD.

We found a positive correlation between sleep difficulties and problematic mealtime behaviors; furthermore, sleep difficulties were also associated with stronger aggressive and stereotyped behaviors, suggesting that children with worse sleep quality show more challenging behaviors throughout the day. Several studies highlight the importance of investigating sleep disturbances in children with autism. As reported by Cohen et al. [46], sleep is an essential aspect of adaptive functioning, such as learning, memory, and neuroplasticity, so sleep deficiencies may worsen symptoms of ASD, in particular by exacerbating difficult behaviors. In addition, research has shown a strong correlation between affective problems and sleep problems among children and adolescents with a diagnosis of ASD. Correlations have also been identified between affective problems and daytime sleepiness, sleep duration, and sleep anxiety [47]. Furthermore, previous literature studies have shown that co-occurring conditions can have an impact on challenging behaviors in individuals with a diagnosis of ASD, and these conditions can be linked to psychiatric conditions [48].

We also found a positive relationship between sleep disorders and parental stress. This finding deserves particular attention; in fact, it has been reported that high levels of parental stress could affect the success of behavioral interventions dedicated to the child. Therefore, screening for sleep problems should be included when parents seek assistance to improve their stress levels and child and family care. This is because evidence from previous studies suggests that child sleep problems are associated with poorer parental mental health and higher parenting stress [49].

As a secondary aim, we examined the frequency and type of GI and feeding disorders through a systematic in-hospital multidisciplinary assessment that included a pediatric gastroenterological evaluation. Feeding disorders and GI symptoms are frequently reported as comorbidities in children with ASD, but the prevalence rates vary greatly among different studies in the literature, ranging from 9% to 91% [10,11,50]. This wide variability could depend on the different methods used to gather and assess the symptoms, as most of the studies rely on questionnaires or parents’ reports only.

In line with previous findings, functional constipation was the most common GI disorder in our sample of children with a diagnosis of ASD (36%). A recent review of the literature identified a median prevalence range for constipation of 22% (range 4.3–45.5%) in ASD children [11]; Kang et al. revised clinical data from 164 children with ASD evaluated at a pediatric neurology practice and reported that 49% had one or more chronic GI complaints, with 26% suffering from constipation [26].

Gorrindo et al. [15] reported a high frequency of functional GI disorders in a sample of ASD children assessed by a physician’s evaluation compared to non-ASD children, with functional constipation being the most common diagnosis in the ASD-GID group (85% of the diagnoses), followed by GERD. However, the exact frequency of constipation in the ASD sample was not reported.

FS is the most reported atypical feeding behavior in ASD [18], and it was present in nearly 60% of children in our sample. In 10 (28%) cases, FS was associated with moderate to severely restricted food repertoire and food refusal. These data are consistent with previous observations. Schreck et al. surveyed 472 caregivers of children between 5 and 12 years of age and reported that 72% of children with ASD had feeding difficulties, a significantly higher rate than typically developing same-age peers [51].

In our study, weight assessment through growth charts showed that most children (80%) had a normal weight; however, being overweight or obese was more frequent than being underweight. This is consistent with previous observations [51] and could be related to multiple factors, including reduced physical activity and food selectivity with a food repertoire limited to energy-dense foods. Raspini et al. showed that Italian preschoolers with ASD consumed significantly higher amounts of simple sugars, processed and ultra-processed carbohydrates, and lower amounts of vegetables and fruits compared to peers without ASD and reported an obesity rate of 6.2%, more than fourfold as compared to typically developed children [52]. The use of elimination diets, such as gluten-free or casein-free diets, and complementary alternative medicine is common in families with ASD children [53,54]. In our sample, two families reported following a special diet (lactose-free or gluten-free) without a medical prescription.

We did not find a correlation between GI disorders and autism severity expressed by ADOS CSS. Previous studies in the literature showed similar results. Prosperi et al. [19] investigated the prevalence and type of GI and FS symptoms in 163 preschoolers with ASD and their link with behavioral problems. The authors reported that approximately 40% of children had at least one severe GI symptom or FS. Those with GI symptoms and FS did not differ greatly in performance IQ and autism severity. Still, they scored higher on the scale rating stereotyped and self-injurious behaviors, sleep problems, and anxiety.

Finally, we investigated the feedback from families regarding the proposed multidisciplinary approach and the usefulness of the given counseling. The results show that families felt it was important to have a dedicated assessment with a pediatric gastroenterologist about GI symptoms and food selectivity; moreover, they appreciated the gastroenterologist’s expertise about autism, thus knowing how to approach their children best. Parents reported that the counseling given was useful, although they could not always implement suggestions provided by pediatric gastroenterologists in the home setting.

Our experience confirmed a recent UK survey of over 800 parents and professionals that reviewed parent-delivered behavioral interventions tailored by health professionals and found some interventions helpful but not always sufficient to address feeding problems in children with ASD and neurodisabilities [55]. Parent training is reported to be useful for mild and moderate FS in children with ASD [56,57]. However, we are aware that effective treatment in severe cases of FS requires a more intensive behavioral intervention delivered by a trained ABA therapist, with direct meal observations in a dedicated setting.

The study also has several limitations, including the small sample size that is unrepresentative of all children with ASD. In addition, our sample consists mainly of toddlers and young children, so it would be necessary to replicate this study with a wider age range. Additionally, our sample was composed of 55% Italians and 45% other nationalities, which could affect the generalization of results. Moreover, we are conscious that this study describes the sample at the baseline without a follow-up evaluation; a gastroenterological follow-up was scheduled only for children with moderate to severe FS. In this sample subgroup, we observed that some families experienced difficulties addressing feeding problems in the home setting.

This study has some points of strength. First, the multidisciplinary approach with coordinated teamwork of different pediatric specialists (gastroenterologists, child neuropsychiatrists, and psychologists) allowed us to assess the complex interaction between ASD comorbidities better. Second, the actual frequency and type of GI and feeding disorders were assessed through a clinical evaluation by pediatric gastroenterologists, who also received dedicated training to address feeding issues based on ABA principles. Moreover, we used specific questionnaires dedicated to the ASD population (i.e., the CSHQ revised, used as a screening for the presence of sleep problems, GISSI-17 used as a screening for the presence of GI symptoms, and the BPI used to assess the frequency and intensity of behavioral problems). Finally, we believe that the novelty of this study is that every child had a visit with a pediatric gastroenterologist, who received dedicated training to address feeding problems based on ABA principles, rather than just relying on validated questionnaires given to the children’s parents. Compared with previous studies [25,26], we believe that the added value of this study is the use of validated questionnaires specific to children with autism and the inclusion of specialist visits with both a neuropsychiatrist and a pediatric gastroenterologist.

## 5. Conclusions

In conclusion, our data indicate that gastrointestinal and feeding disorders, sleep problems, and challenging behaviors are common comorbidities in children with a diagnosis of ASD and can exacerbate each other. Specifically, in our sample, aggressive behaviors were more prevalent in children with more sleep difficulties, and this association was stronger in children who had more negative mealtime experiences. Additionally, sleep disorders were found to be correlated with stereotyped behaviors and parent-perceived stress.

The findings of this study highlight the importance of an integrated assessment of gastrointestinal problems, challenging behaviors, and sleep disorders in children on the ASD spectrum. These data suggest that children may benefit from multidisciplinary assessments involving trained specialists to diagnose comorbidities and provide targeted advice to parents. Further research should explore the relationship between these issues in a larger sample of children on the autism spectrum to evaluate the effectiveness of the proposed best practices and to investigate and treat severe cases of feeding disorders with targeted behavioral interventions in specialized settings.

## Figures and Tables

**Table 1:** Socio-demographic and clinical characteristics.

Variables	Means (SD) or Number (%)
*Sex*	
Male	29 (81%)
Female	7 (19%)
*Age*	
Children’s age (years)	4,5 (2.2)
Age range 0–3 years	11 (30%)
Age range 3–6 years	18 (50%)
Age range 6–10 years	7 (19%)
*Nationality*	
Italian	20 (55%)
Others	16 (45%)
*ASD Severity*	
ADOS CSS	4,4 (3.6)
*Maternal Educational Level*	
Middle School	8 (22.2%)
High School	12 (33.3%)
University degree or higher	8 (22.2%)
Other/Missing	8 (22.2%)
*Paternal Educational Level*	
Middle School	3 (8.3%)
High School	17 (47.2%)
University degree or higher	6 (16.6%)
Other/Missing	10 (27.7%)
*Maternal Occupational Status*	
Employed	13 (36.1%)
Unemployed	15 (41.6%)
Other/Missing	8 (22.2%)
*Paternal Occupational Status*	
Employed	26 (72.2%)
Unemployed	0 (0%)
Other/Missing	10 (27.7%)

**Table 2:** Means, standard deviations, and correlations with confidence intervals.

Variable	*M*	*SD*	Age	BMI	CSHQ	BAMBI	CSS ADOS	BPI Aggressive	BPI Autolesive	BPI Stereotype
1. Age	4.56	2.25								
2. BMI	18.20	4.24	0.37 [−0.16, 0.73]							
3. CSHQ	45.46	7.77	0.13 [−0.21, 0.44]	0.03 [−0.47, 0.52]						
4. BAMBI	43.65	12.21	0.34 * [0.01, 0.61]	−0.07 [−0.56, 0.46]	0.41 * [0.09, 0.66]					
5. CSS ADOS	6.36	2.51	−0.01 [−0.40, 0.39]	0.25 [−0.41, 0.74]	0.01 [−0.38, 0.41]	−0.02 [−0.41, 0.38]				
6. BPI Aggressive	5.06	7.27	0.16 [−0.18, 0.47]	−0.34 [−0.71, 0.19]	0.50 ** [0.20, 0.71]	0.51 ** [0.21, 0.72]	−0.26 [−0.59, 0.15]			
7. BPI Autolesive	3.89	5.02	0.24 [−0.10, 0.53]	−0.34 [−0.72, 0.18]	0.31 [−0.02, 0.59]	0.46 ** [0.15, 0.69]	−0.01 [−0.40, 0.39]	0.71 ** [0.49, 0.84]		
8. BPI Stereotype	22.29	18.46	0.14 [−0.20, 0.46]	−0.17 [−0.61, 0.36]	0.50 ** [0.20, 0.71]	0.52 ** [0.21, 0.73]	−0.04 [−0.43, 0.36]	0.77 ** [0.58, 0.88]	0.74 ** [0.54, 0.86]	
9. PSI total score	68.90	33.01	0.41 * [0.07, 0.67]	0.22 [−0.38, 0.69]	0.47 ** [0.14, 0.71]	0.15 [−0.23, 0.48]	0.05 [−0.38, 0.46]	0.24 [−0.12, 0.55]	0.33 [−0.03, 0.61]	0.30 [−0.06, 0.59]

Note. M and SD are used to represent mean and standard deviation, respectively. Values in square brackets indicate the 95% confidence interval for each correlation. The confidence interval is a plausible range of population correlations that could have caused the sample correlation (Cumming, 2014). * Indicates *p* < 0.05. ** indicates *p* < 0.01.

**Table 3:** Linear regression models with PSI scores as dependent variables.

	PSI Total Score			PSI_PD		
*Predictors*	*b*	*95% CI*	*p*	*b*	*95% CI*	*p*
Intercept	66.56	55.30–77.81	**<0.001**	51.43	38.12–64.74	**<0.001**
BAMBI	−10.95	−24.85–2.95	0.117	−14.18	−30.59–2.23	0.087
CSHQ	18.15	6.96–29.35	**0.003**	23.67	10.35–36.99	**0.001**
Age	20.38	6.64–34.11	**0.005**	21.08	5.14–37.02	**0.012**
BAMBI × CSHQ	5.34	−5.56–16.24	0.323	7.05	−5.88–19.98	0.273
R^2^		0.432			0.426	

Note. Estimates b have been computed after predictors have been standardized.

**Table 4:** Linear regression models with BPI scores as dependent variables.

	BPI Aggressive			BPI Autolesive			BPI Stereotype		
*Predictors*	*b*	*95% CI*	*p*	*b*	*95% CI*	*p*	*b*	*95% CI*	*p*
Intercept	4.17	2.06–6.29	**<0.001**	3.51	1.81–5.22	**<0.001**	22.06	16.29–27.83	**<0.001**
BAMBI	1.03	−1.52–3.58	0.417	1.01	−1.04–3.07	0.322	4.61	−2.34–11.57	0.185
CSHQ	3.34	1.13–5.56	**0.004**	1.17	−0.62–2.95	0.191	7.70	1.66–13.73	**0.014**
Age	1.36	−1.01–3.74	0.250	1.33	−0.58–3.24	0.165	2.65	−3.83–9.13	0.409
BAMBI × CSHQ	2.82	0.68–4.95	**0.011**	1.34	−0.37–3.06	0.120	2.67	−3.14–8.49	0.355
R^2^		0.501			0.321			0.411	

Note. Estimates b have been computed after predictors have been standardized.
